# Emotional and sleep problems and their associated factors among specialized operation personnel on surface vessels

**DOI:** 10.1186/s40359-026-04930-7

**Published:** 2026-06-12

**Authors:** Guorui Liu, Yanfei Zhang

**Affiliations:** 1 Department of Medical Psychology, Second Affiliated Hospital of Naval Medical University, Shanghai, 200003 China; 2 Department of Medical Psychology, No. 905 Hospital of PLA Navy, Shanghai, 200052 China

**Keywords:** Surface vessels, Specialized operation, Depression, Anxiety, Sleep

## Abstract

**Objective:**

To investigate the prevalence of anxiety, depression, and sleep problems among specialized operation personnel on surface vessels, and to analyze their influencing factors.

**Methods:**

A total of 600 participants were randomly selected. Basic demographic and occupational information was collected using a general information questionnaire. The Self-Rating Anxiety Scale (SAS), Self-Rating Depression Scale (SDS), and Pittsburgh Sleep Quality Index (PSQI) were administered to assess anxiety, depression, and sleep quality.

**Results:**

Spearman correlation analysis indicated that anxiety symptoms was associated with years of service (*r* = 0.130, *P* = 0.001), family residence (*r* = -0.153, *P* < 0.001), and sleep quality (*r* = 0.199, *P* < 0.001). Depression symptoms was associated with parental relationship (*r* = -0.134, *P* = 0.001), family residence (*r* = -0.144, *P* < 0.001), and sleep quality (*r* = 0.122, *P* < 0.05). Binary logistic regression further showed that PSQI was associated with anxiety (OR = 2.187, *P* = 0.001). All PSQI subscales were correlated with SAS and SDS total scores (*P* < 0.05).

**Conclusion:**

Sleep and emotional problems are prevalent among specialized operation personnel on surface vessels. Greater attention should be given to the psychological health of subgroups at higher risk, such as older individuals, those with rural backgrounds, and those with longer years of service. Routine psychological screening is recommended for this population.

With the growing global attention to mental health, the psychological well-being of individuals in special occupations has become a focus of public and academic concern [1, 2]. Personnel in specialized operations on surface vessels face considerable psychological stress and challenges due to the unique nature of their working environment and occupational demands.

In the present study, “specialized operation personnel” refers to crew members on surface vessels engaged in long-distance maritime missions, responsible for critical operational and technical tasks. These personnel differ from other maritime roles as they focus on sustained operational support during extended offshore voyages, rather than routine coastal duties. Their responsibilities include handling high-risk tasks, making decisions under pressure, and responding to emergencies, often in confined spaces and under prolonged stress [3]. In such settings, personnel are required not only to endure high-intensity physical and cognitive labor but also to cope with disrupted circadian rhythms, limited social support systems, and the additional burden of responding to emergencies. Collectively, these factors increase the risk of developing mental health problems [4].

Research from chronopsychology and psychophysiology has shown that disrupted sleep patterns can negatively affect mental health, with sleep disturbances contributing to heightened anxiety, depression, and overall emotional dysregulation in both general populations and specific occupational groups. International studies across various maritime and isolated high-stress occupations have shown a consistent trend in the prevalence of depression, anxiety, and sleep disturbances. For instance, studies have reported that depression affects between 10 and 30% of personnel in specialized maritime operations, while anxiety rates range from 15 to 25% across different countries [5–6]. Additionally, sleep disorders are commonly found in approximately 25–40% of workers in maritime and isolated environments, with poor sleep quality being linked to significant emotional and cognitive impairments. These findings align with the global prevalence rates observed in similar high-risk, high-demand occupations.

Recent studies have illustrated that poor sleep quality and insufficient sleep are strongly linked to the dysregulation of key brain regions involved in emotion regulation, such as the prefrontal cortex and amygdala, leading to heightened emotional reactivity and reduced capacity to cope with stress [7]. Sleep deprivation has been found to increase the activity of the hypothalamic-pituitary-adrenal (HPA) axis, which governs the body’s stress response, making individuals more vulnerable to emotional distress and psychological disorders [8, 9]. Moreover, chronic insomnia and irregular sleep-wake cycles have been associated with the development of mood disorders, including major depressive disorder and generalized anxiety disorder, as they disrupt the natural circadian rhythms that play a key role in regulating mood and cognitive performance [10, 11]. Conversely, poor mental health can exacerbate sleep disturbances, leading to a vicious cycle of psychological and physiological challenges [12]. For instance, depression and anxiety can increase the hyperarousal states that interfere with the ability to initiate and maintain sleep, with individuals experiencing racing thoughts, heightened alertness, and physical tension, all of which disrupt sleep onset and sleep continuity [13]. This bi-directional relationship underscores the complex interplay between sleep and mental health, as psychological distress can increase the likelihood of developing sleep disorders, while sleep problems in turn worsen mental health conditions, creating a feedback loop that is difficult to break. Research has shown that this cycle is particularly pronounced in high-stress occupational settings, where individuals are exposed to significant work-related demands, social isolation, and environmental stressors that disrupt both sleep and mental well-being.

International comparisons have further highlighted that personnel in high-stress and isolated environments, such as those on long-distance voyages, exhibit similar psychological and sleep disturbances to those seen in shift workers, emergency responders, and healthcare professionals, all of whom face disruptions to their circadian rhythms and prolonged social isolation.Previous studies have reported that seafarers exhibit higher prevalence rates of depression, anxiety, and sleep disorders compared to the general population. Such conditions can impair work efficiency, reduce attention and response times, and increase the likelihood of operational errors and accidents, thereby posing serious risks to both vessel safety and crew health [14–16]. The associations between sleep and mental health in seafarers may mirror those observed in other high-stress, isolated populations, such as shift workers and emergency responders, who face similar challenges related to circadian rhythm disruption and social isolation. Although research on the mental health of surface vessel personnel has increased in recent years, studies on the psychological well-being of personnel performing specialized operations during long-distance voyages remain relatively scarce. In particular, the interactive mechanisms between emotional states and sleep disturbances in these specialized personnel have been underexplored [17]. Compared with short-term coastal navigation, long-distance voyages involve extended periods away from home, greater environmental uncertainty, and more severe physiological and psychological adaptation challenges, highlighting the urgent need for focused investigation into this population’s mental health.

In this study, we employed standardized psychometric assessments in combination with interviews to investigate the prevalence and severity of anxiety, depression, and sleep disturbances among personnel during operational periods, and to identify associated influencing factors. The findings aim to provide scientific evidence and theoretical guidance for the prevention of mental health and sleep problems in this specialized population. Furthermore, this research seeks to inform the development of targeted psychological interventions and health promotion strategies, ultimately enhancing both the mental well-being and operational safety of these personnel.

## Methods

### Participants

From October to December 2023, a total of 600 personnel engaged in shift work during long-distance maritime operations on a surface vessel were randomly selected as study participants. Mid-voyage, participants underwent on-site psychological assessments using the SAS, SDS, or PSQI. This study was designed as a cross-sectional observational study following the STROBE reporting guidelines.

### Measures

#### General information questionnaire

Demographic and occupational variables were collected, including sex, age, years of service, education level, marital status, number of children, spousal relationship, relationship with parents, family residence, family upbringing environment, status as an only child, and family history of mental illness among close relatives. 

#### Self-Rating Depression Scale (SDS) [18, 19]

The SDS consists of 20 items, each rated on a 4-point Likert scale (1 = rarely or none of the time; 2 = some of the time; 3 = a considerable part of the time; 4 = most or all of the time). Ten items (2, 5, 6, 11, 12, 14, 16, 17, 18, 20) are reverse scored. The raw score is summed and then multiplied by 1.25 to yield the standard score. A standard score of 53 serves as the cutoff: 53–62 indicates mild depression, 63–72 indicates moderate depression, and > 72 indicates severe depression. Cronbach’s alpha: 0.88–0.93. 

#### Self-Rating Anxiety Scale (SAS) [20, 21]

The SAS also consists of 20 items rated on the same 4-point scale. Five items (5, 9, 13, 17, 19) are reverse scored. The raw total score is multiplied by 1.25 to generate the standard score. A score of 50 is the threshold: 50–59 indicates mild anxiety, 60–69 moderate anxiety, and > 69 severe anxiety. Cronbach’s alpha: 0.87–0.93. 

#### Pittsburgh Sleep Quality Index (PSQI) [22]

The PSQI consists of 19 items covering seven domains: subjective sleep quality, sleep latency, sleep duration, sleep efficiency, sleep disturbances, use of sleep medication, and daytime dysfunction. Each domain is scored from 0 to 3, with higher scores indicating poorer outcomes. The total score ranges from 0 to 21, with > 7 indicating poor sleep quality.

### Sample size calculation

Based on an expected medium effect size (*r* = 0.3 for correlation analysis, Cohen’s f² = 0.35 for logistic regression), a statistical power of 0.80, and a significance level of α = 0.05, the required sample size was calculated to be 350 participants. This sample size was deemed sufficient to detect significant correlations and to ensure robust results for the logistic regression analysis. To account for potential non-responses and data attrition, a larger sample size was recruited. In addition, various sources of bias were controlled for during the analysis. Selection bias was minimized by employing random sampling, ensuring a representative sample of the target population. Measurement bias was reduced by using validated, standardized tools (SAS, SDS, and PSQI), with all participants receiving standardized instructions during data collection. Information bias was minimized through anonymous data collection and by providing participants with clear information about the study and its confidentiality measures. A total of 600 participants were included in the final analysis, ensuring adequate power and reliability of the study findings while reducing the risk of bias.

### Category merging criteria

To ensure the robustness and stability of the statistical models, we merged categories based not only on low frequencies but also on a conceptual justification. Categories with frequencies below 5% of the total sample size were considered for merging to avoid issues related to sparse data, which can lead to extreme or undefined odds ratios in logistic and ordinal regression models. Additionally, categories with fewer than 10 participants were combined with conceptually similar categories to maintain adequate sample sizes, thereby reducing the risk of model overfitting and enhancing the generalizability of the results.

The conceptual justification was based on identifying categories that shared comparable demographic or behavioral characteristics, ensuring that merged categories remained theoretically coherent. The comparability of merged categories was further evaluated using Chi-square or Fisher’s exact tests, depending on sample size, to ensure that the combined groups were statistically reasonable.

By applying these criteria, we aimed to improve model stability, address the limitations associated with sparse data, and maintain both the theoretical coherence and interpretability of the demographic categories.

### Statistical analysis

Data were analyzed using SPSS version 26.0. Categorical variables were expressed as frequencies and percentages, and continuous variables were expressed as (mean ± standard deviation). Correlations between anxiety, depression, and sleep quality were analyzed using Spearman’s test. Logistic regression was performed to identify associated factors. Two-tailed tests were applied, and *P* < 0.05 was considered statistically significant.

## Results

### Sample characteristics

A total of 600 surface specialized operation personnel were included in this study. All participants independently completed the questionnaires on site, with 600 questionnaires distributed and 600 valid questionnaires returned, yielding a response rate of 100%. Detailed demographic information, including age, sex, and educational level, is presented in Table 1.

**Table 1:** Descriptive Characteristics of the Sample

Item	*N* (%)
Age (Years)	
< 25 Years	414 (69.0)
≥ 25 Years	186 (31.0)
Gender	
Male	412 (68.7)
Female	188 (31.3)
Years of Work Experience	
< 2 Years	176 (29.3)
≥ 2 Years	424 (70.3)
Educational Level	
Junior college or below	468 (78.0)
Bachelor’s degree or above	132 (22.0)
Marital Status	
Unmarried and Divorced	465 (77.5)
Married	135 (22.5)
Children Status	
Has Children	70 (11.7)
No Children	530 (88.3)
Relationship with Parents	
Average and Good	92 (15.3)
Very Good	508 (84.7)
Family Residence	
Rural	300 (50.0)
Town and County	165 (27.5)
Urban	135 (22.5)
Family Growth Environment	
Complete Family	541 (90.2)
Incomplete Family	59 (9.8)
Only Child	
Yes	226 (37.7)
No	374 (62.3)
Family History of Mental Illness	
Yes	0 (0)
No	600 (100)
SAS	
<50points	509 (84.8)
50-59points	78 (13.0)
60-69points	12 (2.0)
≥ 70points	1 (0.2)
SDS	
<53points	400 (66.7)
53-62points	144 (24.0)
63-72points	55 (9.2)
≥ 73points	1 (0.2)
PSQI	
≤ 7points	380 (63.3)
> 7points	220 (36.7)

### Factors associated with depression, anxiety, and sleep quality

As shown in Table 2, anxiety was significantly associated with years of work experience (*r* = 0.130, *P* = 0.001), family residence (*r* = − 0.153, *P* < 0.001), and sleep quality (*r* = 0.199, *P* < 0.001). Depression was associated with parental relationship (*r* = − 0.134, *P* = 0.001), family residence (*r* = − 0.144, *P* < 0.001), and sleep quality (*r* = 0.122, *P* < 0.05). The total PSQI score was associated with years of work experience (*r* = 0.126, *P* = 0.002), SAS score (*r* = 0.383, *P* < 0.001), and SDS score (*r* = 0.271, *P* < 0.001), but not with age (*r* = 0.034, *P* = 0.409).

**Table 2:** Analysis of Related Factors for Depression, Anxiety, and Sleep Conditions

Item	SAS(≥ 50)		SDS(≥ 53)		Total PSQI Score	
	*r*-value	*p*-value	*r*-value	*p*-value	*r*-value	*p*-value
Gender	-0.019	0.647	-0.064	0.115	0.034	0.409
Age	0.061	0.136	0.006	0.886	0.096	0.018^*^
Years of Work Experience	0.130	0.001^**^	0.067	0.100	0.126	0.002^**^
Educational Level	-0.030	0.457	-0.069	0.091	0.002	0.967
Marital Status	0.065	0.109	0.040	0.330	0.080	0.051
Children Status	0.020	0.625	0.029	0.473	0.051	0.210
Relationship with Parents	-0.074	0.072	-0.134	0.001^**^	0.056	0.173
Family Residence	-0.153	0.000^***^	-0.144	0.000^***^	0.034	0.412
Family Growth Environment	-0.063	0.122	-0.028	0.498	0.043	0.289
Whether an Only Child	0.051	0.216	0.075	0.065	0.020	0.623
PSQI≥7Points	0.199	0.000^***^	0.122	0.003^**^		
Total SAS Score					0.383	0.000^***^
Total SDS Score					0.271	0.000^***^

*SAS* Self-Rating Anxiety Scale, *SDS* Self-Rating Depression Scale, *PSQI* Pittsburgh Sleep Quality Index

* < 0.05,** < 0.01, *** < 0.001

To improve the stability of the regression model, categories with small sample sizes in some variables (e.g., educational level, marital status) were collapsed into broader groups. Ordinal logistic regression revealed that personnel with a rural upbringing had a 3.384-fold higher likelihood of experiencing anxiety compared with those raised in urban settings (OR = 3.606, *P* = 0.001). Good sleep quality was associated with a significantly lower risk of anxiety (OR = 0.314, *P* < 0.001). The goodness-of-fit tests for the regression models were performed using Pearson χ^2^ and Deviance χ^2^ tests. The Pearson χ^2^ = 111.521 (df = 145), *P* = 0.982. Similarly, the Deviance χ^2^ = 80.802 (df = 145), *P* = 1.000 (Table 3). Similarly, rural upbringing was associated with a higher risk of depression (OR = 2.164, 95% CI: 1.321–3.560, *P* = 0.002), while good sleep quality was associated with a reduced risk of depression (OR = 0.530, *P* < 0.001). The Pearson χ^2^ = 124.924 (df = 145), *P* = 0.885. Similarly, the Deviance χ^2^ = 119.876 (df = 145), *P* = 0.937 (Table 3).

Binary logistic regression analysis of PSQI-related factors indicated that anxiety (SAS) exerted a greater impact on sleep quality than depression (OR = 2.187, *P* = 0.001) (Table 4). The correlation between the SDS and PSQI approaches statistical significance (OR = 1.302, *P* = 0.086).

**Table 3:** Ordinal Logistic Regression Analysis of the Determinants of Anxiety and Depressive Symptoms

Independent Variables	SAS		SDS	
	OR (95% CI)	*p*-value	OR (95% CI)	*p*-value
Sex (Male vs. Female)	1.395 (0.156, 12.758)	0.766	4.901 (0.53, 45.08)	0.160
Age (≤ 25 years vs. >25 years)	1.033 (0.530, 2.026)	0.927	1.146 (0.680, 1.919)	0.604
Years of Work Experience (< 2 years vs. ≥2 years)	0.519 (0.256, 1.057)	0.071	0.733 (0.463, 1.159)	0.184
Educational Level (Junior college or below vs. Bachelor’s degree or above)	0.973 (0.538, 1.759)	0.927	1.427 (0.902, 2.260)	0.131
Marital Status (Unmarried and Divorced vs. Married)	0.480 (0.221, 1.037)	0.064	0.647 (0.353, 1.197)	0.170
Children Status (With Children vs. Without Children)	1.468 (0.607, 3.595)	0.393	0.864 (0.431, 1.721)	0.677
Relationship with Parents (Average and Relatively Good vs. Very Good)	1.556 (0.855, 2.841)	0.149	2.429 (1.548, 3.806)	< 0.001^***^
Family Residence (Rural vs. Urban)	3.606 (1.719, 7.564)	0.001^**^	2.164 (1.321, 3.560)	0.002^**^
Family Residence (Township and County vs. Urban)	1.567 (0.735, 3.342)	0.287	1.744 (1.021, 2.963)	0.040^*^
Family Growth Environment (Incomplete Family vs. complete Family)	2.020 (0.755, 5.463)	0.162	0.930 (0.391, 2.240)	0.877
Whether an Only Child (Yes vs. No)	0.950 (0.564, 1.597)	0.948	0.860 (0.591, 1.258)	0.439
PSQI Score (≤ 7 points vs. >7 points)	0.314 (0.195, 0.507)	0.000^***^	0.530 (0.373, 0.759)	< 0.001^***^

SAS coding: <50 points = 0, 50–59 points = 1, 60–69 points = 2, > 69 points = 3.SDS coding: <53 points = 0, 53–62 points = 1, 63–72 points = 2, > 72 points = 3. SAS: Self-Rating Anxiety Scale; SDS: Self-Rating Depression Scale; PSQI: Pittsburgh Sleep Quality Index. PSQI Score (≤ 7 points = 1, > 7 points = 2); Sex (Male = 1, Female = 2);Age (≤ 25 years = 1, > 25 years = 2); Years of Work Experience (< 2 years = 0, ≥ 2 years = 1); Educational Level (Junior college or below (High School and Junior College) = 1, Bachelor’s degree or above (Bachelor’s Degree and Graduate and Above) = 2); Marital Status (Unmarried and Divorced = 1, Married = 2); Children Status (With Children = 1, Without Children = 2); Relationship with Parents (Average and Relatively Good = 1, Very Good = 2); Family Residence (Rural = 1, Township and County = 2, Urban = 3); Family Growth Environment (Incomplete Family = 1, Complete Family = 2); Whether an Only Child (Yes = 1, No = 2)

Frequency Distributions: Sex: Male = 412 (68.7%), Female = 188 (31.3%); Age: <25 years = 414 (69%), ≥ 25 years = 186 (31%); Years of Work Experience: <2 years = 176 (29.3%), ≥ 2 years = 424 (70.3%); Educational Level: Junior college or below = 468 (78.0%), Bachelor’s degree or above = 132 (22.0%); Marital Status: Unmarried and Divorced = 465 (77.5%), Married = 135 (22.5%); Children Status: With Children = 70 (11.7%), No Children = 530 (88.3%); Relationship with Parents: Average and Relatively Good = 92 (15.3%), Very Good = 508 (84.7%); Family Residence: Rural = 300 (50.0%), Township and County = 165 (27.5%), Urban = 135 (22.5%); Family Growth Environment: Incomplete Family = 59 (9.8%), Complete Family = 541 (90.2%); Whether an Only Child: Yes = 226(37.7), No = 374(62.3); PSQI Score: ≤7 points = 380 (63.3%), > 7 points = 220 (36.7%)

* < 0.05,** < 0.01,*** < 0.001

**Table 4:** Binary Logistic Regression Analysis of Factors Influencing Sleep Status

Independent Variables	OR (95% CI)	*p*-value
Sex	1.756 (0.470, 6.587)	0.412
Age	1.289 (0.742, 2.082)	0.344
Years of Service	1.630 (0.763, 1.892)	0.256
Educational Level	1.007 (0.731, 1.288)	0.965
Marital Status	0.898 (0.291, 2.344)	0.841
Children Status (Yes/No)	2.310 (0.655, 2.637)	0.135
Relationship with Parents	0.792 (0.487, 1.050)	0.252
Family Residence	1.115 (0.961, 1.289)	0.152
Family Growth Environment	1.298 (0.610, 1.299)	0.646
Whether an Only Child	0.919 (0.648, 1.366)	0.660
SAS	2.187 (1.608, 4.695)	0.001^**^
SDS	1.302 (0.759, 1.758)	0.086

PSQI Score (≤ 7 points = 1, > 7 points = 2)

*SAS *Self-Rating Anxiety Scale, *SDS* Self-Rating Depression Scale, *PSQI* Pittsburgh Sleep Quality Index

** < 0.01

### Correlation of depression, anxiety, and sleep factors

As presented in Table 5, all PSQI components were significantly correlated with both SAS and SDS total scores (*P* < 0.05). The correlations between SAS and PSQI factors were stronger than those between SDS and PSQI factors in six domains: subjective sleep quality, sleep latency, sleep duration, sleep disturbances, use of sleep medication, and daytime dysfunction.

**Table 5:** Correlation Analysis of PSQI Factors and SAS, SDS Total Scores among Naval Officers and Sailors

Item	Total SAS Score		Total SDS Score	
	*r*-value	*p*-value	*r*-value	*p*-value
Sleep Quality	0.340	0.000^***^	0.245	0.000^***^
Sleep Latency	0.261	0.000^***^	0.151	0.000^***^
Sleep Duration	0.234	0.000^***^	0.134	0.001^**^
Sleep Efficiency	0.122	0.003^**^	0.129	0.002^**^
Sleep Disturbance	0.286	0.000^***^	0.211	0.000^***^
Use of Sleep Medications	0.137	0.001^**^	0.136	0.001^**^
Daytime Dysfunction	0.258	0.000^***^	0.184	0.000^***^

** < 0.01, *** < 0.001

All participants who scored positively on the SDS and SAS (indicating mild to severe depression and anxiety) confirmed the presence of corresponding symptoms during the interviews. The interview findings revealed that the severity of depression and anxiety in these individuals ranged from mild to severe. However, no quantitative analysis was conducted on the interview data.

## Discussion

This study found that 36.7% of specialized operation personnel on surface vessels experienced sleep problems. Additionally, 33.3% reported depressive symptoms, and 16.2% reported anxiety symptoms. Compared with previous studies, the proportion of sleep problems in this population has now surpassed that of emotional problems.

Studies have shown that surface personnel engaged in specialized operations exhibit higher scores on depression and anxiety scales compared to their counterparts in land-based specialized occupations, with prevalence rates approaching 30% [23, 24]. Studies on risk factors have suggested that older age, being an only child, and a tendency toward ruminative thinking are associated with greater vulnerability to psychological problems [25, 26]. The present study revealed similar prevalence rates of depression (33.3%) and anxiety (16.2%) compared with earlier findings [27]. Specifically, depression was associated with poor parental relationships and poor sleep quality, whereas anxiety was associated with longer years of service and poor sleep quality. Interestingly, while previous research reported that personnel raised in urban environments were more prone to emotional problems [25], the current findings suggest that those with a rural upbringing may have a higher likelihood of experiencing depressive and anxiety symptoms. It should be emphasized that these findings reflect associations rather than causal or developmental relationships. Earlier studies often emphasized that rural-raised specialized operation personnel were more resilient, obedient, and better able to endure hardship, although their potential psychological vulnerabilities have received less attention [26].

Since the 1980s and 1990s, large numbers of rural residents migrated to urban areas for work, resulting in a substantial population of “left-behind children.” From the social-ecological perspective, prolonged separation from parents during childhood has been linked to negative self-concept, perceived discrimination, and loneliness [28–31]. Left-behind children were typically raised by grandparents, who may have limited ability to provide psychological support. Combined with weaker rural educational resources and higher peer-bullying risk, these factors may be associated with a higher likelihood of depression and anxiety among personnel with rural backgrounds [32–34].

Previous studies have indicated that sleep problems are highly prevalent among surface specialized operation personnel, severely impairing their operational capacity [3, 35]. Environmental factors include confined living spaces, poor ventilation, high temperature and humidity, noise, and vessel motion. Occupational factors include long voyages, frequent shift work, job burnout, and limited psychological resilience [3, 22, 36]. Reported prevalence rates of sleep disturbances vary widely from 15% to 65% [18, 35]. In the present study, the prevalence of sleep problems was 36.7%, falling within this range and supporting the reliability of our findings.

Research on factors influencing sleep quality among surface-operation personnel has produced inconsistent results. Some studies have reported associations between sleep problems and voyage duration, anxiety, and depression [37], but no correlation with age, education, or years of service [38]. Others have found links with marital status, years of service, emotional states, and education level [39]. In addition, shift schedules during voyages have been identified as an objective determinant of sleep quality [36, 40]. In the present analysis, PSQI scores were correlated with age, years of service, and both SAS and SDS scores, partially consistent with earlier findings. While some studies suggested a correlation between education level and sleep quality [40], no such association was found here, warranting further comparative research. Marital status approached statistical significance (*P* = 0.051), suggesting that larger sample sizes may help clarify these associations.

Prior studies have also reported associations between sleep disturbances and negative emotions [41–43], However, regression analyses indicated that anxiety had a stronger association with sleep quality than depression, consistent with anxiety being a major insomnia risk factor [7, 44]. Neuroimaging studies further suggest overlapping neural pathways and neurotransmitter systems between sleep and anxiety, involving structures such as the limbic system (amygdala, insula, anterior cingulate cortex) and hippocampus [44]. These shared mechanisms may underlie their bidirectional relationship, although the precise neurobiological basis requires further research. Some studies reported that anxiety primarily affects sleep efficiency and daytime function, whereas our findings indicate both anxiety and depression influence sleep comprehensively, including onset, maintenance, and daytime functioning [45, 46]. Moreover, subjective sleep quality—a core PSQI component—showed the strongest correlations with depressive and anxiety symptoms [42, 47].

## Conclusion

In summary, surface specialized operation personnel experience a high prevalence of sleep problems as well as depressive and anxiety symptoms. These rates are comparable to those reported in previous studies. By incorporating a broader range of demographic and psychosocial variables, our study indicates that rural upbringing may be associated with higher likelihood of depression and anxiety in this population. These findings highlight the need for targeted interventions and systematic approaches to support mental health. Sleep quality was significantly influenced by age and years of service, and strongly correlated with both anxiety and depression. These results underscore the importance of monitoring and improving sleep and psychological well-being among surface-operation personnel.

This study has several limitations. First, the reliance on self-reported questionnaires may introduce recall bias, which could affect the accuracy of the data on sleep problems and emotional symptoms. Second, the study did not stratify participants based on voyage duration, and future research should include data from different mission durations to enable more nuanced comparative analyses. Third, the cross-sectional design limits causal inference, providing only a snapshot of associations between sleep and emotional symptoms. Residual confounding may also exist, as unmeasured variables or interactions could partly explain observed associations. Despite controlling for a range of potential confounders, there may still be unmeasured variables or unaccounted-for interactions that could explain some of the observed associations. This is a common challenge in observational studies, and future research should attempt to address this issue by incorporating longitudinal data or more comprehensive control variables. In terms of regression instability, while we have taken steps to minimize overfitting by reducing the number of predictors and categories, despite the simplification procedures and category merging implemented to enhance model stability, a residual risk of regression instability and overfitting cannot be entirely ruled out. Future studies could employ cross-validation or sensitivity analyses to better assess model stability. Lastly, potential biases related to sampling or measurement could also affect the generalizability of the results. For instance, the sample may not be fully representative of the broader population, and the methods used to measure emotional symptoms and sleep disturbances may not capture all relevant dimensions of these complex constructs.

## Data Availability

All relevant data are presented in the text and the tables.
